# DNA extraction from a maize (*Zea mays* L.) seed without damaging germination ability

**DOI:** 10.5511/plantbiotechnology.24.1122a

**Published:** 2025-03-25

**Authors:** Jae-Hong Kim, Ji Won Kim, Minah Jung, Gibum Yi

**Affiliations:** 1 Department of Bio-Environmental Chemistry, College of Agriculture and Life Sciences, Chungnam National University, 99 Daehak-ro, Yuseong-gu, Daejeon 34134, Republic of Korea

**Keywords:** corn, DNA extraction, genotyping, germination, maize seeds

## Abstract

DNA extraction with reliable purity and concentration is essential for most of the molecular genetics studies. Extracting DNA from young leaves in seedling stage is advantageous because it causes less damage to remaining plant which can be further used for phenotypic analysis. DNA extraction from seeds is even more advantageous in terms of saving time, labor, space, and cost for germination. Maize is one of the most important food and feed sources and provides great materials for genetic and breeding studies which are accompanied by genotyping and phenotyping. We present seed DNA extraction method which does not cause damage the seed’s germination ability. DNA was extracted using cetyltrimethyl-ammonium bromide method or a commercial DNA extraction kit from the seed fragment, and the quantity and quality of the DNA were examined. Seed germination was tested for proportional seed cuts at 0, 10, 30, and 50% of the distal end of a seed, proportionally by weight. Extracting DNA from the distal seed fragments resulted in high-quality and sufficient amount of DNA. Germination rates were not significantly reduced when seed cuts were made at 10 or 30% of seed weight. DNA extraction from seeds cut can be an efficient way to obtain samples for genotyping and phenotyping. Moreover, it can be applied for high-throughput DNA extraction in maize and possibly to other smaller seeds.

Genomic DNA extracted from various tissue samples is widely utilized for DNA sequencing, PCR-based genotyping, and other molecular techniques (Kesawat and Das Kumar 2009). Efficient preparation of high-quality DNA is crucial for plant genetic studies. Plant DNA extraction is largely performed in a leaf-based genotyping system, which involves planting candidate genotype plants in the field, sampling leaf tissue from the plants, and conducting genotyping to identify plants with the desired phenotype (Gao et al. 2008). However, a seed-based genotyping system offers an alternative that can save resources and enhance the efficiency of molecular breeding applications. By extracting DNA from seeds, genotype analysis can be conducted before planting, thereby eliminating the complex process of leaf sampling and saving resources, field space, and labor (Zheng et al. 2015). Seed-DNA extraction thus provides significant savings and convenience for both genotyping and phenotyping.

DNA extraction from grain seeds such as rice, barley, and maize has been previously reported (Abdel-Latif and Osman 2017; Mutou et al. 2014; von Post et al. 2003). Seed tissues containing endosperm and cotyledons can be used to extract DNA (Galli et al. 1986). Even though the endosperm has a triploid genome and is composed of starch granules, it has been shown to be sufficient for DNA extraction (Yi et al. 2011).

However, there are some limitations. Seed germination is essential for obtaining next-generation plant materials and conducting phenotypic analysis. If seeds are damaged during DNA extraction, germination cannot occur, hindering further research. Additionally, seeds are usually small and dried tissues, making it relatively difficult to obtain sufficient quantity and quality of DNA (Sudan et al. 2017). Seed DNA extraction without causing damage is therefore efficient, allowing for simultaneous genotyping and phenotyping (Gao et al. 2008).

Maize is one of the most important food and feed sources and serves as a model for genetic and biological research (Coe 2001; Ranum et al. 2014). Marker-assisted selection is widely used in breeding industries and genetics laboratories, and a lab-scale high-throughput maize seed DNA extraction method has also been reported (Gao et al. 2008). In this study, we investigated the amount of seed tissue required to obtain sufficient DNA without compromising the seed germination rate. We determined cutting range of a maize seed that provides adequate samples for DNA extracting without affecting the seed’s germination ability. This method could be applied to high-throughput DNA extraction in maize and potentially in other smaller seeds such as rice and wheat, thereby enhancing efficiencies for genetic and breeding research.

An F1 hybrid cultivar, ‘Sinhwangok’ (Son et al. 2017), five inbred lines (Icheon Chal, Paju Geukjo, Dent t1, Hongcheon Chal, and Inje Chal), eleven maize germplasm (genebank.rda.go.kr (Accessed Dec 30, 2024)) were used in this study (Kim et al. 2023). The maize seeds were either harvested from the nursery at Chungnam National University farm (Daejeon, Korea) or kindly provided by the National Institute of Crop Science, Rural Development Administration (Suwon, Korea). All the seeds were harvested in 2021 or 2022, and used in the experiments in 2022. Randomly selected seeds were cut and divided with a razor blade into distal and proximal parts based on seed weight (0, 10, 30, and 50%).

The germination test was conducted in the greenhouse for 7 days. The test was performed by repeating it three times with each of 100 seeds, including thirty uncut and seventy cut seeds cut by weight at 10% (30 ea), 30% (30 ea), and 50% (10 ea). Each treatment was seeded on trays with 8×5 holes with mixed soil media, and the germination rate was determined by appearance of seedling above ground and recorded every day. For the field germination test, 15×3 uncut and 15×3 cut seeds that were cut by 10% of their weight were used. The germination rate was recorded every day for a week after sowing seeds directly into soil.

Genomic DNA was extracted from the distal parts of the seeds using two methods: the cetyltrimethyl-ammonium bromide (CTAB) method and a commercial DNA purification kit based on silica adsorption (Abdel-Latif and Osman 2017). CTAB extraction was performed at room temperature as described by Doyle (Doyle 1991) with some modifications. A seed fragment, a portion of the distal part cut off, was soaked in 250 µl of CTAB buffer for 5 min and ground with plastic pestle attached to a hand-held drill. Additional 250 µl of CTAB buffer was added and mixed vigorously. The sample was centrifuged at 12,000 rpm for 5 min. The supernatant was transferred to a new tube without disturbing the pellet containing seed coat and starch granules. The supernatant was mixed with 250 µl of chloroform and centrifuged at 12,000 rpm for 10 min. The supernatant (about 250 µl) was transferred to a new tube, 50 µl of 7.5 M ammonium acetate and 500 µl of ice-cold absolute ethanol were added to precipitate the DNA. The mixture was then centrifuged at 12,000 rpm for 10 min. The supernatant was discarded and the pellet was recovered. For washing the pellet 500 µl of 70% ethanol was added and mixed. The pellet was recovered again, sufficiently dried to remove residual ethanol, and dissolved in 100–200 µl of sterilized distilled water. The sample was then stored at 4°C. DNA separation by silica adsorption was performed using the DNeasy plant mini kit according to the manufacturer’s protocol (QIAGEN, Germany).

DNA concentration was measured using a NanoDrop 1000 UV/Vis spectrophotometer (Thermo Scientific, USA) at 260 nm (A260) absorption, while purity was assessed by estimating the absorption ratios A260/A280 and A260/A230. The quality of the extracted DNA was also evaluated by 0.8% agarose gel electrophoresis.

The PCR reaction solution was composed to a final volume of 10 µl, containing 10–20 ng of template DNA, 10 pmole each of forward and reverse primers, 5 µl of 2× GoTaq master mix (Promega, USA), and distilled water to reach 10 µl. The thermal cycling conditions were as follows: pre-incubation at 94°C for 5 min; followed by 30 cycles of 94°C for 30 s, 55°C for 30 s, 72°C for 30 s; with a final extension step of 5 min at 72°C. SSR markers used for PCR were selected from Maize Genetics and Genomics Database (Table 1; http://www.maizegdb.org (Accessed Dec 30, 2024)). PCR products were electrophoresed on 1.2% agarose gel using 0.5× TBE buffer at 100 V for 25 min.

**Table 1:** Table 1. SSR markers used in this study.

SSR marker	Repeat motif	Forward primer	Reverse primer	Product size (bp)	Tm (°C)	Bin No.
umc2246	(CCTCCT)_4_	aggctccagctctaggggagt	gtgaactgtgtagcgtggagttgt	146	69	2.00
umc1024	(GA)_8_	cctttttcgcctcgctttttat	tcgtcgtctccaatcatacgtg	172	64	2.04
umc1229	(AG)_12_	aaacttctcccccgcagttc	caccaactccaccacgttcc	219	68	6.01
umc1152	(ATAG)_6_	ccgaagataaccaaacaataatagtagg	actgtacgcctccccttctc	171	63	10.02

*Detailed information is available at www.maizegdb.org

To check the duration of storability at room temperature, the cut seeds were stored for 0, 1, 4, and 8 weeks. To determine whether the coating of the section affects the germination rate, a half of seeds were coated with the tree wound protector ‘Lac Balsam (ETISSO, Germany)’. Thirty coated and thirty uncoated seeds after cutting 10% of the weight were stored for 0 to 8 weeks, and their germination ability was tested under greenhouse conditions.

The distal ends of maize seeds were cut off at 0, 10, 30, and 50% of their individual seed weight to determine the range of seed cutting that allows for high-quality DNA extraction without affecting germination ability (Figure 1). The distal fragments of the cut seeds were used for DNA extraction, and the proximal parts were planted to assess germination and viability. No physical damage on embryo was observed in any seeds cut by 10% of their seed weight, while slight or significant damage was observed in embryos cut by 30 and 50%, respectively. The injured parts of the embryo were mostly scutellum.

**Figure 1:**
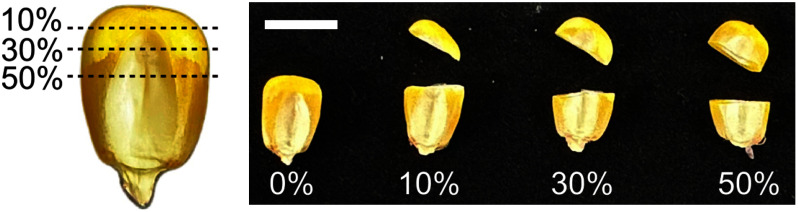
Figure 1. Seed cutting rates by weight. Maize seeds were cut into two pieces according to the percentage of weight (distal end/total). Bar=1 cm.

The germination ratio was not significantly different in 0, 10, and 30% cut seeds, while 50% cut seed made a significant reduction in germination ratio (Figure 2A). The germination rate of the 50% cut seeds began to significantly decrease from the 3 days after planting, and reached 85% of maximum at 5 days after planting (DAP), while the germination rates of other treatment groups reached 100% (Figure 2A). The height of the seedlings germinated from cut seeds was observed on 7 days after planting. Compared to seedlings from 0% and 10% cut seeds, those from seeds cut by 50% displayed a significant decrease in plant height (Figure 2B). Since the endosperm provides energy for germination and growth during the early stages of seed germination, plant height tends to decrease as the seed cutting ratio increases, as shown by the linear regression (R^2^=0.5238, *p*=2.492×10^−9^) analysis (Figure 2C). The damage to the embryo in the 50% cutting seeds restricted the continuous growth of the seedlings. At 14 DAP, while the growth of all seedlings in the other treatment groups was maintained, only half of the seedlings in the 50% cutting group continued to grow.

**Figure 2:**
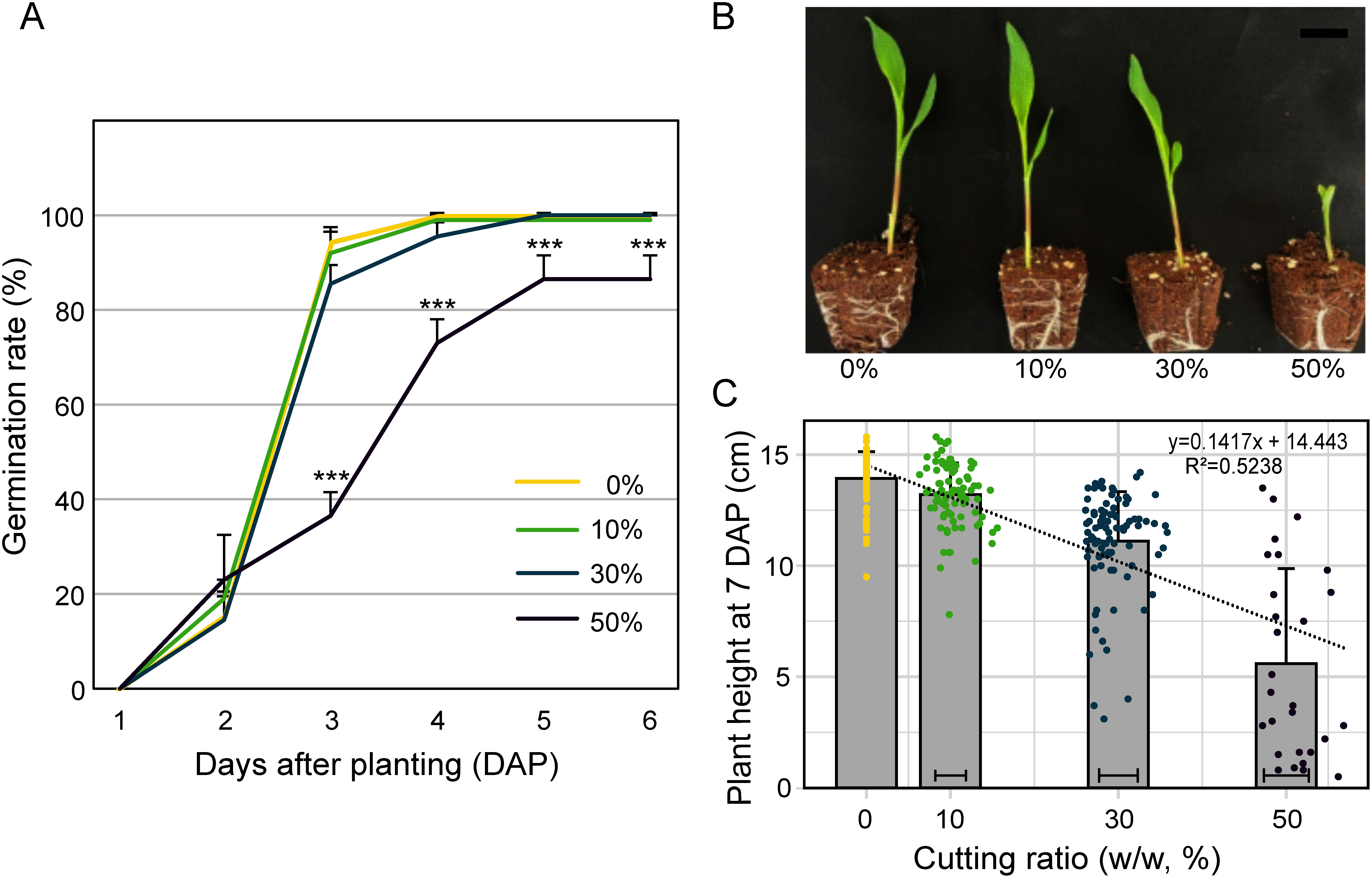
Figure 2. Germination rate and plant height at 7 DAP depending on the ratio of seed cut. (A) Germinated individulas were counted for 6 days after planting. *** indicate *p*<0.001 which is based on Tukey HSD after one-way ANOVA. (B) Representative plants germinated from 0, 10, 30, and 50% cut seeds (from left to right). Scale bar=3 cm. (C) Plant heights were measured at 7 DAP plants presented with mean±SD and plotted by their cutting ratio and plant height. Horizontal error bars inside the each bar graph indicate stand deviations for each cutting ratio.

To determine the optimal range of seed fragment necessary for obtaining sufficient quality DNA for genotype analysis, genomic DNA was extracted from the distal part of maize seeds. Two different methods were used for DNA extraction; CTAB method, and commercial column-based DNA extraction.

The amount of DNA was increased proportionally to the weight of seed tissue used for extraction (Figure 3). In addition, DNA extracted with the DNeasy plant mini kit provided good A260/280 (1.57) values. However, DNA extracted using the CTAB method had protein contamination with A260/280 values ranging from 0.74 to 1.10.

**Figure 3:**
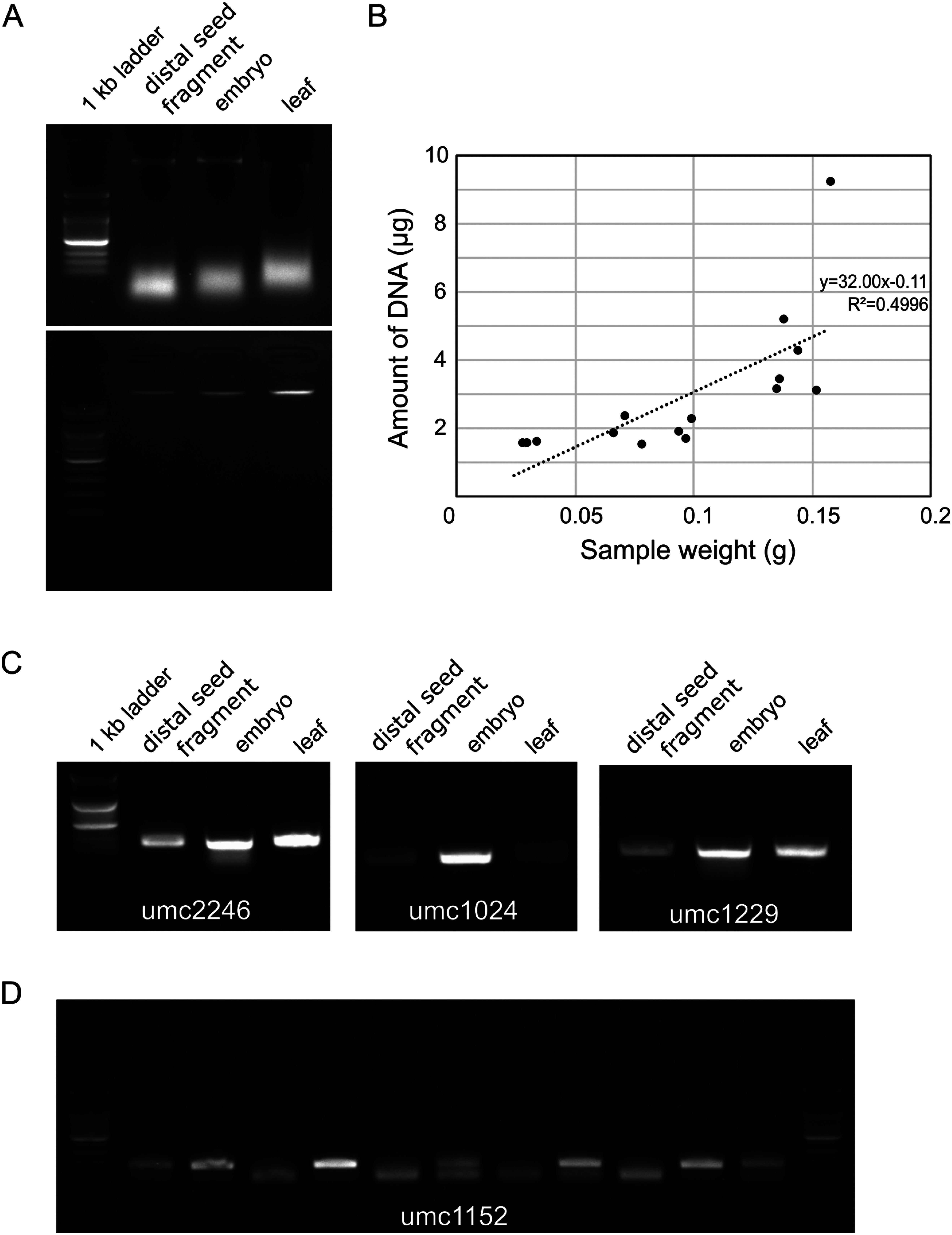
Figure 3. Verification of DNA extraction from distal ends of maize seeds. (A) Agarose gel image of DNA extracted with CTAB method (upper pannel, without RNase treatment) commercial DNA extraction kit (lower pannel, with RNase treatment) from different tissues. (B) Amount of DNA extracted from distal ends of seeds with different weight. (C) PCR amplification with the extracted DNA from different tissues. (D) Practical application of a single seed cut DNA for SSR genotyping. Genomic DNA was extracted by CTAB method from the cut seed of the following 11 maize germplasm (IT112909, IT162789, IT62790, IT04497, IT101254, IT103942, IT104707, IT104788, IT105182, IT105171, IT134951; from genebank.rda.go.kr (Accessed Dec 30, 2024)) and then genotyped with the umc1152 SSR marker.

The extracted seed DNA was evaluated for the feasibility of PCR analysis with SSR markers. It was compared to DNA extracted from leaves and embryos. DNA extracted from distal parts, embryos, and germinated young leaves produced clear bands with similar intensity (Figure 3A). The CTAB DNA extraction method without RNase treatment showed RNA bands at the bottom, which were removed by RNase treatment. Furthermore, DNA obtained from the distal parts of the seed was good enough for PCR analysis (Figure 3C). DNA extracted from the diverse germplasm seeds displayed both homozygote and heterozygote bands, demonstrating its potential utility for effective genotyping for hybrid and inbred lines (Figure 3D). The simple CTAB DNA extraction method, even without RNase treatment, was adequate for determining the genotypes with SSR markers.

Five inbred lines including dent, and waxy types were also used to confirm that a non-destructive sampling method was applicable for wide variety of maize. Inbred seed was tending to be smaller than the F1 hybrid seed. There was no significant difference in germination rate between uncut and 10% cut seeds, showing seed DNA extraction method is applicable in most of the maize varieties (Figure 4).

**Figure 4:**
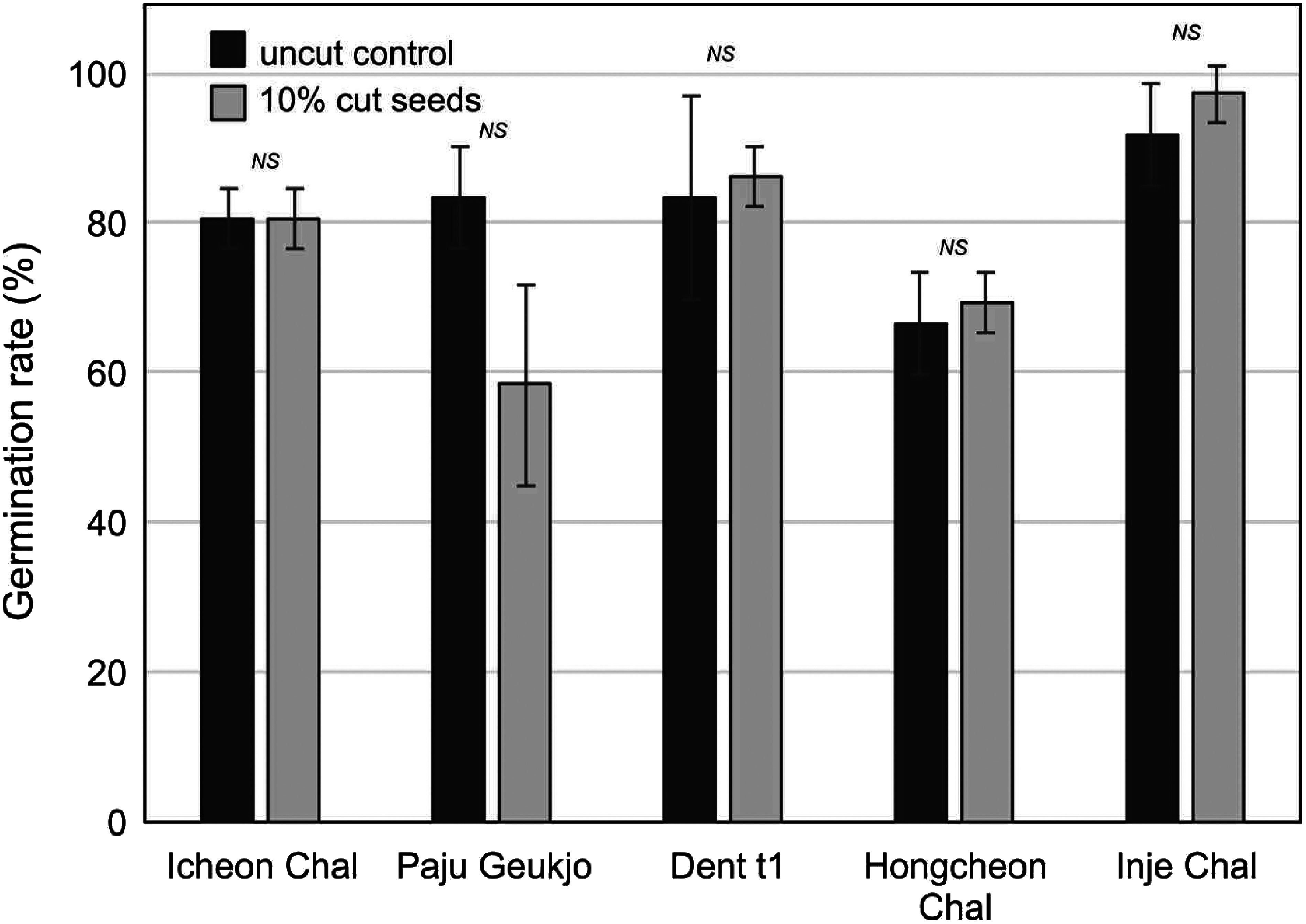
Figure 4. Application of the seed cut method on five inbred lines. Dent and waxy maize inbreds were used and 10% cut seeds showed no significant difference in their germination rate compared to uncut control. ‘*NS*’ indicates no significant difference by Student’s *t*-test.

Maize seeds can be stored for several years at room temperature. Cut seeds can be easily dried or cause contamination into the cut sections over time, affecting germination. Therefore, after sampling to extract DNA, a germination test was performed after storage at room temperature for 1, 2, 4, and 8 weeks, respectively. The germination ability of 10% cut seeds was not reduced until at least two months, the germination rate was retained to be more than 85% (Figure 5A). It should be noted that the unharmed embryos in these 10% cut seeds contribute to retaining their germination ability. Germination may have been affected by the infiltration of pathogens or fungi on the cross section of maize seeds when it was planted in soil. We applied surface coating with commercial wound protecting material and there was no significant difference in their germination rate (Figure 5B). We could not find any effect of wound-protecting material in this experiment, but it could be considered to help maintain seed germination ability for a longer period and under harsher conditions.

**Figure 5:**
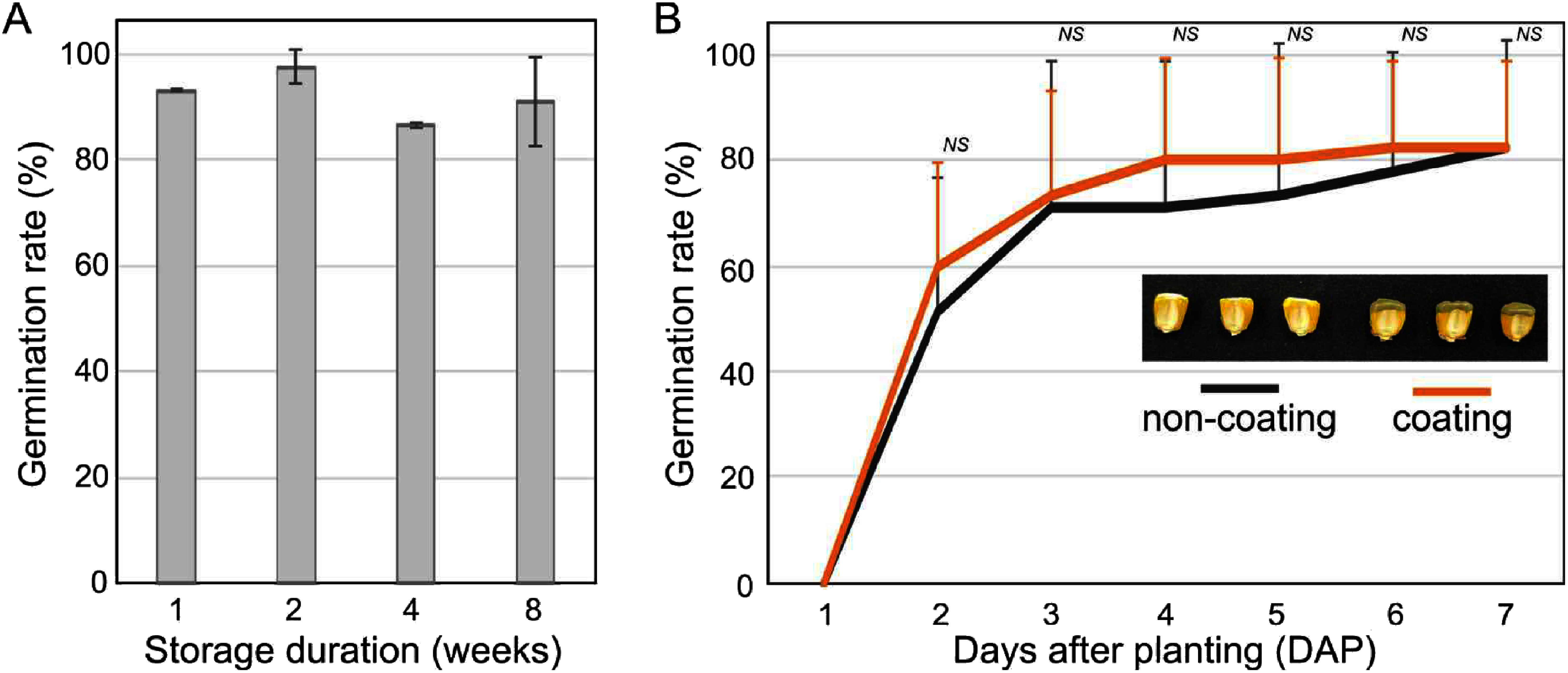
Figure 5. Effects of storage duration and seed coating of cut seeds on germination rate. (A) 10% cut seeds were stored for 1–8 weeks at room temperature and tested for their germination rate. (B) 10% cut seeds were coated with wound protector and compared their germination rate with non-coating control on field condition. ‘*NS*’ indicates no significant difference between coated and non-coated cut seeds according Student’s *t*-test.

A non-destructive sampling method is essential for seed-based genotyping systems in molecular breeding programs. A number of methods have been introduced to extract DNA from seeds for crops such as rice (Liang et al. 2016), soybean (Kamiya and Kiguchi 2003), and maize (Gao et al. 2008). However, during seed sampling, seeds are often deformed or damaged, making it difficult to germinate and obtain healthy plants from the remaining seeds. Therefore, in this study, we tested seed cutting range in maize seeds to ensure both successful DNA extraction and germination.

The endosperm provides energy for seed germination and early seedling growth (Lopes and Larkins 1993). After sampling, the remaining seeds have less energy than uncut seeds, resulting in reduced germination rates and decreased plant height during the seedling stage (Figure 2A, B). Meru reported similar results in watermelon seed samples; when the seeds were cut by 30%, the germination rate was 95%, but when cut by 50%, the germination rate dropped to 43% due to damage to the embryos (Meru et al. 2013).

Seed samples for DNA extraction consisted of the pericarp and endosperm (Halilu et al. 2013). Although the endosperm’s triploid genome can lead to different genotyping results compared to diploid plants, the heterozygote genotype showed only slight differences in PCR band intensity without changing the genotype itself (Yi et al. 2011). DNA was extracted in all samples in which the seeds were cut from 10 to 50%, yielding is in the range of 2.16–13.16 µg of DNA, sufficient for hundreds to thousands of PCR reactions (Figure 3B). The DNA quality can be affected by the high starch composition in endosperm according to the extraction methods, but it did not affect the PCR analysis (Chunwongse et al. 1993).

This method offers an alternative for selecting suitable individuals prior to planting and facilitates analysis in small labs lacking sufficient field space to grow crops. It will be applicable to other seeds and will increase efficiency in breeding and genetics studies.

Here we showed that a 10–30% distal maize seed cut sample by weight is sufficient for obtaining genomic DNA and safe for germinating the remaining seeds. Different DNA extraction methods including rapid and high-throughput protocols can be applied with the seed cut samples. The cut seeds can be stored at room temperature at least for two months and coating of the cut surface may enhance storability of the seeds. Thus, DNA extraction and germination are possible at the same time with this method.
